# Receptors That Inhibit Macrophage Activation: Mechanisms and Signals of Regulation and Tolerance

**DOI:** 10.1155/2018/8695157

**Published:** 2018-02-11

**Authors:** Ranferi Ocaña-Guzman, Luis Vázquez-Bolaños, Isabel Sada-Ovalle

**Affiliations:** Laboratorio de Inmunología Integrativa, Instituto Nacional de Enfermedades Respiratorias “Ismael Cosío Villegas”, Ciudad de México, Mexico

## Abstract

A variety of receptors perform the function of attenuating or inhibiting activation of cells in which they are expressed. Examples of these kinds of receptors include TIM-3 and PD-1, among others that have been widely studied in cells of lymphoid origin and, though to a lesser degree, in other cell lines. Today, several studies describe the function of these molecules as part of the diverse mechanisms of immune tolerance that exist in the immune system. This review analyzes the function of some of these proteins in monocytes and macrophages and as well as their participation as inhibitory molecules or elements of immunological tolerance that also act in innate defense mechanisms. We chose the receptors TIM-3, PD-1, CD32b, and CD200R because these molecules have distinct functional characteristics that provide examples of the different regulating mechanisms in monocytes and macrophages.

## 1. Introduction

### 1.1. Macrophages

Macrophages are phagocytic cells, which are localized through the whole human body. Monocytes give rise to these terminally differentiated cells. Monocytes and macrophages belong to the functional immune system known as mononuclear phagocyte system which includes dendritic cells, circulating monocytes, and their progenitors in bone marrow.

Macrophages have several functions to maintain immune homeostasis such as host protection, tissue repair, phagocytosis, clearance, and secretion of diverse factors, which contribute to innate and adaptive defenses against infection and counteract inflammatory processes, while distinct secreted signals restore tissue homeostasis and promote subsequent repair [1, 2]. To perform protective functions and repair damaged tissue, monocytes and macrophages express a wide range of surface, vacuolar and cytosolic receptors for recognition, and uptake of host-derived (damage signals) and foreign particles; many of these receptors facilitate phagocytosis, endocytosis, sense viral, bacterial, and parasitic molecules [3].

During organogenesis, macrophages expressed by the embryonic yolk sac and fetal liver remain as a resident cell, self-maintaining population, which turn over locally under steady-state conditions and perform a variety of clearance and organ-specific trophic functions [4]. After birth, bone marrow-derived blood monocytes replenish resident macrophage populations with high turnover rate, such occurs in the gut; larger numbers are recruited following injury, infection, and sterile inflammation and give rise to infiltrating and activated tissue macrophages [5].

Depending on the anatomical localization and its organ requirements, macrophages consist of variably mixed populations of resident macrophages and blood-derived monocytes. As a result of their complex origin, distribution, and physiologic responses to endogenous and exogenous stimuli, these cells will express a marked phenotypic heterogeneity (reviewed by Gordon and Plüddemann) [6].

Macrophages and monocytes are characterized by a multifunctional heterogeneity. For example, macrophages can be polarized in two main subtypes, “classical” and “alternative” activated macrophages. Classical macrophages depend on the presence of proinflammatory cytokines such as INF-*γ* and TNF-*α* secreted by activated TH1 CD4^+^ lymphocytes and NK cells and secreted bacterial components such as LPS. Classical macrophages are also known as M1 which has enhanced antimicrobial, inflammatory, and antigen-presenting properties [7]. Alternative macrophages are generated in presence of IL-4 and IL-13 cytokines secreted by activated TH2 CD4^+^ lymphocytes. These macrophages play an important role limiting inflammatory responses, perform antiparasitic functions, and favor wound healing [8]. Moreover, alternative macrophages now can be classified in a new proposed scheme M2 like M2a, M2b, and M2c subtypes with specific functions and markers (reviewed by Martinez and Gordon) [8].

Activation through the surface, vacuolar, and cytosolic receptors results in signals to control or regulate functions in their neighbors and distant target cells. Their phagocytic capacity is variable and may even be undetectable but provides a well-developed machinery to internalize, degrade, and store cargo such as poorly degraded foreign particles [9].

Also, macrophages expressing regulatory surface molecules can attenuate or inhibit cell activation, which could be considered a tolerance or compensation mechanism as a result of an exacerbated immune response, so they have to preserve tissue homeostasis to avoid an inflammatory process which can compromise the homeostasis.

### 1.2. Inhibitory Receptors

Cells of the immune system are activated by endogenous or exogenous antigenic stimuli. Endogenous stimuli are often causal agents of autoimmune diseases but can also come from transformed cells (cancer cells). Exogenous stimuli include a broad range of environmental compounds, as well as other molecules that may come from pathogens like bacteria, fungi, parasites, and viruses that have entered into the organism where they can, potentially, establish infections [10]. These stimuli activate diverse cellular mechanisms whose purpose is to prevent or eliminate infections that are frequently accompanied by inflammatory processes. It is now well-accepted that the activation of the immune system is a process highly regulated through the expression and functioning of inhibitory receptors that work to prevent an exacerbated inflammatory response that can cause tissue damage or even autoimmune diseases.

Surface receptors are proteins that extend through the cell membrane. Their function is to transduce signals into the cell in response to some external stimuli. Receptors have intracellular domains that associate with cytoplasmic proteins such as adaptors, chaperone proteins, or enzymes which are required to perform adequately signaling. These receptors contain diverse conserved domains such as ITAM, ITIM, and ITSM. ITAM is perhaps the one that has been described in greatest detail. ITAM domains are found in several receptors, including T cell receptors (TCR), B cell receptors (BCR), and Fc region receptors (FcR). Studies of the structure of ITAM domains have identified a conserved sequence characterized by the presence of two tyrosine residues (Y), each one followed by two variable residues and one residue of leucine in the Y + 3 position, which is considered as a conserved domain that can trigger activation signals through conformational changes and phosphorylation of tyrosine residues, as well as the direct or indirect association with enzymatic elements present in the cytoplasm [11, 12]. Receptors with ITAM domains are associated with cell activation in response to stimuli or the ligands of those receptors. However, another group of receptors exists with the function of moderating cell activation. These are the inhibitory receptors.

The concept of the inhibitory receptor was introduced early in the 1990s [13, 14]. These receptors have been defined as molecules that negatively regulate the immune response to pathogenic microorganisms [15] and contain ITIM domains in the intracellular tail that, when phosphorylated, recruit enzyme phosphatases such as SHP1 and SHP2, which interfere with the activation pathways promoted by other activator receptors [16]. ITIM domains are similar to ITAM domains in that both have a specific arrangement of tyrosine/leucine [17]. This tyrosine/leucine arrangement in inhibitory receptors is located inside of a sequence of 13 amino acid residues that usually have a hydrophobic residue at position −2 [18].

Recent publications have demonstrated that inhibitory receptors may, or may not, have ITIM domains in the cytoplasmic tail [19]. Other mechanisms have also been described that allow these receptors to regulate activation of cells in the immune system. They include recognition and binding to ligands that perform the function of costimulatory molecules. In this way, inhibitory receptors block the cell from receiving the signals necessary for cell activation in response to specific antigens. In addition to recruiting enzyme phosphatases and competing for ligands, they can also be considered as a mechanism that regulates the status of cell activation/inhibition [20]. In general, inhibitory receptors can interfere in diverse stages of cell activation mediated by antigenic stimuli, inhibit the expression of genes involved in cell activation, and possibly induce the expression of other genes that inhibit the function characteristic of cell activation to produce deleterious changes at the level of cell metabolism, proliferation, and survival [21].

Therefore, we can define inhibitory receptors as molecules of the cell surface that interfere in various ways with intracellular signaling pathways to negatively regulate cell activation and cell function in response to tumors, infections, allografts, and even allergens and many other antigens. Numerous studies have described these receptors based on the diverse mechanisms of tolerance and anergy present in T lymphocytes and NK cells [22–26].

## 2. Receptors That Inhibit Macrophage-Monocyte Functions

### 2.1. PD-1 Belongs to the Superfamily of Immunoglobulins, and Its Function Is Associated with Cell Death and Regulating the Activation of Distinct Cell Types

**Figure 1:**
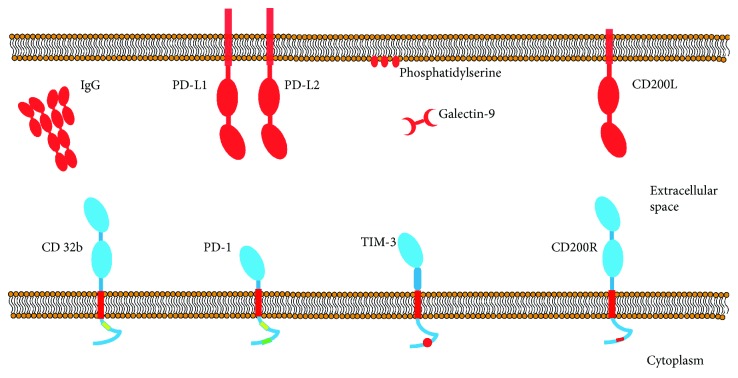
Schematic representation of the structures of inhibitory receptors. All receptors belong to the superfamily of immunoglobulins. They have a single transmembrane portion and an intracellular tail, through which they associate with proteins or effector enzymes. The immunoglobulin domains are represented as blue ovals for receptors and red ovals for ligands.

PD-1 (CD-279) is an inhibitory protein made up of 288 amino acids from the superfamily of the immunoglobulins. Initial studies were associated with assays on cell death in the 1990s [27]. Since then, we have learned that PD-1 is a protein whose expression is induced during the apoptosis process or after administration of apoptotic stimuli. Once synthetized, it is found in the cell membrane. The structure of PD-1 contains ITIM domains and an immunoreceptor tyrosine-based switch motif (ITSM) (Figure 1) [27]. Because PD-1 is a homologous protein to CD28, two ligands belonging to the B7 family were quickly identified: PD-L1 and PD-L2 [28, 29]. The function of PD-1 has been studied widely in T lymphocytes and other lymphoid cells, and it is now well documented that expression of this protein is associated with a dysfunctional state characterized by anergy in the presence of antigenic stimuli, a low rate of proliferation, and reduced cytokine production by PD-1+ cells. This phenotype, in which T lymphocytes express PD-1, is known as the phenotype of “exhausted” lymphocytes, and it has been identified in patients with chronic viral infections and oncological diseases [30, 31]. Blocking with monoclonal antibodies aimed at PD-1 or its ligands, PD-L1, and PD-L2 has been proposed as a therapeutic strategy that could revert the exhausted state of the lymphocytes [32–34].

### 2.1.1. PD-1 Regulates Cell Activation and the Production of Soluble Inflammatory Mediators

One of the mechanisms regulated by PD-1 in macrophages is IL-12 production, as was demonstrated in a group of patients with chronic HCV infection. That study showed that PD-1 expression increased in monocytes in peripheral blood and that this increase was inversely proportional to the production of IL-12 by those cells when compared to healthy subjects or those whose infections had been completely cured [35]. *In vitro* assays revealed that this reduction in IL-12 production is not secondary to the loss of recognition of the virus by the macrophages but, rather, to alterations in the intracellular signaling pathways that include a decrease in the phosphorylation of the JAK/STAT pathway [35]. Treatment with IFN-*α* and ribavirin reduced PD-1 expression, reversed the changes in STAT-1 phosphorylation, and increased production of IL-12 by macrophages in patients infected with HCV [35]. Research using ex vivo experimental models has demonstrated that blocking PD-1/PD-L1 with monoclonal antibodies in samples from patients infected with HCV restores IL-12 production in response to LPS [35, 36]. This mechanism is shared by TIM-3, another inhibitory receptor, since this molecule also negatively regulates TLR-mediated cell activation and IL-12 production [37]. Although these molecules do not belong to the same family, several studies have documented that blocking them is an efficient treatment that makes it possible to restore the activation of cells that are incapable of responding to antigenic stimuli. This was observed first in T lymphocytes [30, 31, 38] and, later, in other cells, including monocytes and macrophages [35, 36]. Despite the fact that the PD-1 molecule was discovered in the early 1990s [27], little is known about its functions in myeloid cells. In another hand, recent studies in the oncologic field have shown that PD-1 is expressed by TAMs in mouse models of cancer and primary human cancer. Gordon et al. demonstrate that PD-1 expression increases over time and with disease stages in humans [39]. Furthermore, PD-1 expression negatively correlates with the phagocytic function to eliminate tumor cells. In this way, these results support the therapy with anti-PD-1 or PD-L1 for distinct kind of tumors [40].

While various studies describe that macrophages and other immune cells express PD-L1 and 2 ligands and, through these molecules, can induce the death of PD-1+ cells [41, 42], the function of PD-1+ macrophages must be investigated deeper in another kind of pathologies such as viral, bacterial, and autoimmune diseases.

### 2.2. TIM-3 Regulates Diverse Functions in Macrophages

#### 2.2.1. Introduction to TIM-3 Signaling and Functions in Macrophages

The TIM-3 protein was initially known as a membrane-specific marker for Th1 and Tc1 lymphocytes [43], but its expression was soon identified in other cell lines. Today, we know that TIM-3 is expressed in monocytes, macrophages, dendritic cells, NK cells, and even diverse cells in different tumor types [31, 44–47]. The extracellular region of TIM-3 consists of a mucin domain and an immunoglobulin domain to which the known ligands of TIM-3 bond, that is, galectin-9 (Gal-9) and phosphatidylserine (Ps) [48, 49]. Glycosylated sites are present in both domains [50] (Figure 1).

Scientific evidence suggests that the interaction of TIM-3 with Gal-9 functions as a negative regulation pathway in cell activation. The absence of this pathway has been associated with the development of autoimmune diseases [40–42]. The way in which signals are transduced into the interior of the cells is via the phosphorylation of tyrosine residues found in the intracellular tail of TIM-3. Currently, we know that there is evidence of the participation of diverse proteins and enzymes in TIM-3 signaling. A model based on the HEK 293 T cell line has shown that TIM-3-mediated signaling begins when the intracellular portion of TIM-3 is phosphorylated by the enzyme interleukin inducible T cell kinase—ITK—from the TEC family of kinases [51]. Phosphorylation by ITK kinase occurs in the tyrosine found at position 265 (Y265) only in the presence of Gal-9 [51]. Another study, this one using the Jurkat, D10, and 293 T cell lines, demonstrated that the Fyn and Lck kinases, which belong to the Src family of kinases, can perform phosphorylation of TIM-3, and further observed that Fyn does so more efficiently [51]. Finally, Lee et al. demonstrated that Bat-3 protein (human leukocyte antigen B-associated transcript 3) is associated with the intracellular portion of TIM-3 in T lymphocytes and that it also recruits a kinase belonging to the Src family (Lck). To date, there are no studies of which kinase participates in TIM-3 phosphorylation when this protein is expressed in monocytes and macrophages. Determining which proteins are involved in the myeloid signaling of TIM-3 could provide scientific knowledge about its function and how it can regulate the different processes identified in this section. Although studies about how TIM-3 function in monocytes and macrophages are scarce, we do know that some mechanisms can be regulated by TIM-3 in these cells in various pathologies, including infectious, autoimmune, and oncological diseases.

Besides being a regulator of activation in macrophages, TIM-3 participates in a process through which damaged cells and apoptotic bodies are removed and eliminated from pluricellular organisms. This mechanism is called efferocytosis [52], a term that refers to the mechanism that works to remove and eliminate cells that have culminated their life cycle or have been damaged by some other biological or physical processes. Some members of the TIM family, including TIM-1, TIM-3, and TIM-4, possess a binding site to phosphatidylserine in the Ig domain, a phospholipid that translocates to the external face of the plasmatic membrane of apoptotic cells and constitutes the principal *eat me* signal, which leads to the capture and elimination of those cells [53]. Crystallography studies have allowed us to determine that TIM-3 binds to phosphatidylserine (PS) through the IgV domain [54, 55]. Since PS is the principle signal for the phagocytosis of apoptotic bodies or cells, blocking recognition of this phospholipid with TIM-3 can induce immunological abnormalities, such as generating autoantibodies, since the detritus of apoptotic cells is not eliminated efficiently [55, 56].

#### 2.2.2. TIM-3 Regulates Cell Activation Via TLR

**Figure 2:**
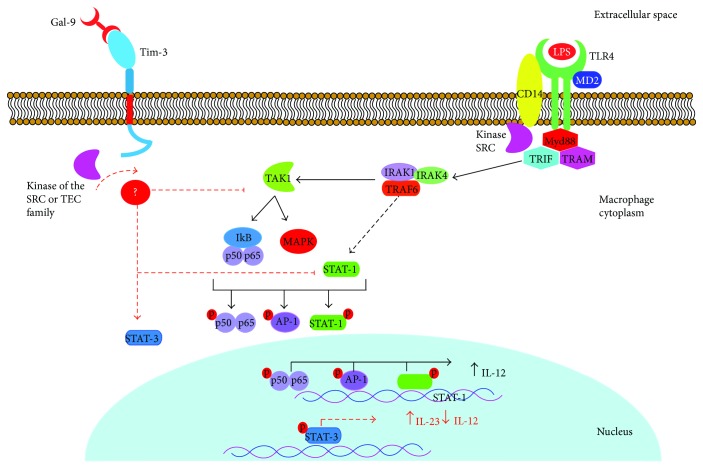
Inhibition mechanism of IL-12 production through interaction of TIM-3 and Gal-9. Graphic representation of the TLR4-signaling pathway (black arrows) when activated by LPS (to simplify, not all proteins involved are shown), which induces phosphorylation of diverse nuclear factors, such as NF-*κ*B (p50-p65), AP-1, and STAT1. These nuclear factors induce production of proinflammatory molecules like IL-12. When TIM-3 interacts with Gal-9, phosphorylation of the intracellular portion of TIM-3 is induced and activates the regulatory pathway mediated by this protein. While we do not yet know the precise inhibition mechanism in macrophages, studies have identified a decrease in STAT1 phosphorylation and an increase in STAT3 phosphorylation (red arrows). The result of this change is reduced IL-12 production and increased IL-23 production.

Research has demonstrated that in addition to regulating Ps-dependent mechanisms, TIM-3 can also regulate macrophage activation and, later, cytokine production when it interacts with Gal-9. Gal-9 is a lectin that recognizes carbohydrates contained in the IgV domain of TIM-3. Based on this interaction, studies have identified that TIM-3 negatively regulates activation of diverse cell types through a mechanism that has been widely studied in T cells [30, 31, 57, 58]. In macrophages, however, little progress has been made in analyzing the process of inhibiting TIM-3-mediated activation. For example, we know that the association of TIM-3 with Gal-9 expressed in other cells (trans), or the macrophage itself (cis), has a distinct effect as a regulator of TLR-mediated activation. The *trans*-association of TIM-3 and Gal-9 negatively regulates TLR-mediated signaling reducing IL-12 production, increasing IL-23 production, and reducing phosphorylation of STAT-1, while also augmenting activation of STAT-3. Meanwhile, the association in Cis fosters corrects TLR signaling, and there is also evidence that the expression of Gal-9 increases through this mechanism [48]. This signaling pathway, which impacts the intracellular proteins STAT-1 and STAT-3, also causes alterations in cytokine production, especially IL-12, since it is solidly documented that IL-12 production is induced only after phosphorylation and translocation to the nucleus of the STAT1 factor. While other nuclear factors such as NF-*κ*B and AP-1 exist and are activated through TLR4, phosphorylation of STAT1 has also been shown to occur, such that the interaction of this nuclear factor with TRAF6 could be involved [59, 60] (Figure 2). Additionally, research has identified that upon silencing TIM-3 expression with siRNA, IL-12 and IL-10 production increases in macrophages derived from the THP-1 line [37, 61].

Together, these data indicate that the expression and function of the TIM-3 receptor negatively regulate IL-12 production and, though to a lesser degree, other cytokines such as IL-10 and IL-6. They further suggest the dynamic character of inhibitory mechanisms and the fine balance between activation and inhibition signals. Therefore, regulation of activation is not restricted to lymphocytes and mechanisms of adaptive immunity in general but is also exerted on innate cells and must be the cause of several pathological disorders that would not have been contemplated in past years.

Although we have not identified all the elements that participate in TLR4-dependent activation, the interaction of TIM-3 with kinases of the Src family must be considered. While several common adapters for different TLR participate in TLR4 activation—such as TRAM, TRIF, and MyD88/MAL—no direct interaction has been found between these elements and any member of the Src family, though interaction between the Src kinase and CD14 has been suggested [62], the latter being an accessory molecule in TLR4 functioning. Also, it has been shown that recognition of LPS by TLR4 is followed by induction of the activation of other members of this family of kinases, including SFK, c-Src, Yes, and Fyn [63]. The fact that the Src family can regulate TLR4-dependent activation as well as TIM-3-mediated inhibition makes analysis more complex and impedes identifying clear signaling pathways for these two processes.

In summary, TIM-3 can simultaneously foster and regulate several important functions in macrophages, including the internalization of apoptotic bodies and activation in response to stimuli captured by TLRs and mechanisms after activation. However, despite the accumulated scientific evidence on the structure and function of TIM-3, it is still not clear how it is that one protein with such distinct functions can act, or exert its effects, on one single cell. In addition to these questions, it seems that the phenomenon of activation/inhibition is being described more often each year. While some reports have documented or proposed possible mechanisms through which TIM-3 fosters the activation of macrophages and lymphocytes, the majority of studies support this protein's role as a negative regulator. This discrepancy may arise from our incomplete understanding of this protein's function and signaling pathway. Given that TIM-3 does not have a conventional ITIM domain, its function does not depend on an association with phosphatases. Hence, the mechanism through which TIM-3 negatively regulates activation pathways in macrophages and T cells must correspond, or perhaps be similar, to that of other inhibitor receptors, like CD200R, which do not require an association with phosphatases to inhibit cell activation. Discovering the precise mechanism that allows TIM-3 to regulate cell activation will propitiate a much better understanding of autoimmune pathologies in whose development the loss of tolerance to autoantigens plays a crucial role. To different degrees, understanding the fine balance between activation and inhibition signals will allow us to develop new therapeutic strategies in diverse areas of immunology.

### 2.3. Fc-Gamma Receptor (CD32b), a Negative Regulator of Macrophage Activation

The family of immunoglobulins includes a heterogeneous group of receptors that can bind to crystallizable regions of immunoglobulins (FcR). The IgG receptors (IgGR) are the ones most widely expressed and may be present in the membrane of most immune cells. According to the functions they perform and the structural motifs they present, these receptors can be classified as activators—Fc*γ*RI, Fc*γ*RIIa, and Fc*γ*RIII—or inhibitors: Fc*γ*RIIb. FcR activators can trigger diverse events in cells that gain expression as increased intracellular Ca^2+^. For these events to occur, the Fc*γ*RI, Fc*γ*RIIa, and Fc*γ*RIII receptors must be associated with accessory chains that possess an ITAM motif and are found in the intracellular tail of receptors, as well as in accessory chains, such as the common gamma chain. Research demonstrates that, like other receptors that have no extensive intracellular tail, Fc*γ*RI and Fc*γ*RIII receptors lose sequences that allow them to associate with proteins or enzymes of the cytosol and so become incapable of mediating signaling on their own once they bond to their ligand. Studies have also shown that the receptors for the Fc region with no extensive intracellular tail can associate with the common gamma chain [64] and, in this way, acquire the capacity to send signals to the interior of the cell. The Fc*γ*RIIa and Fc*γ*RIIb receptors, in contrast, have an intracellular tail with domains that allow them to initiate signaling directly once binding to their ligand occurs, with no need to form an association with accessory molecules. The intracellular tails of these two receptors show substantial changes, and alternative splicing generates more changes in the terminal amino and carboxyl regions of Fc*γ*RIIb [65]. The main differences between the intracellular portions of these molecules are their respective tyrosine-based activation and inhibition motifs.

**Figure 3:**
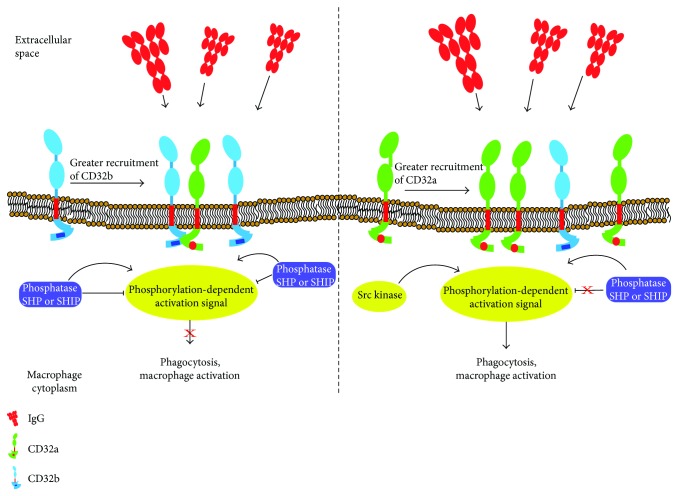
Inhibition mechanism of the Fc*γ*R receptor (CD32). (a) When CD32b recognizes the Fc fraction of IgG, the phosphatase SHP or SHIP is recruited into the ITIM domains of the intracellular portion of the receptor. Those enzymes inhibit phosphorylation of the ITAM domains or eliminate phosphorylation of the ITAM domains contained in CD32a. This mechanism inhibits the activation of macrophages and the phagocytosis of opsonized pathogens and other elements that are susceptible to recognition by IgG-class antibodies. (b) When the association of CD32a is greater than the frequency of CD32b receptors, phosphatase recruitment is not fostered and, therefore, no inhibition of the activation signals mediated by phosphorylation of the ITAM domains in the intracellular portion of CD32b is produced. As a result, the corresponding internalization mechanisms are activated.

In the case of the Fc*γ*RIIb inhibitory receptor, the motif is an off the ITIM type [66] (Figure 1). Although initially the capacity of the Fc*γ*RIIb receptor to modulate activation in a model of B cells was identified [17], we now know that this receptor represents a regulation point for activation that is found in many immune system cells. The mechanism by which the Fc*γ*RIIb receptor performs these functions involves enzymes that eliminate phosphate groups from ITAM domains [67]. The phosphatases usually present in the cytoplasm are SHP (Src homology 2-containing tyrosine phosphatase) and SHIP (Src homology 2-containing inositol phosphatase), which are recruited and associated with the ITIM domain in the Fc*γ*RIIb receptor through the Sh2 domain (Src homology 2) found in these phosphatases [18] (Figure 3). While the FC*γ*RIIb receptor can bond to the enzymes SHP and SHIP, the function of the association with SHIP has been studied more widely in macrophages. The phosphatase SHIP can inhibit signals of cell activation by dephosphorylating phosphatidylinositol triphosphate (PIP3) [68]. In this way, it prevents PIP3-mediated signaling events, such as the translocation of kinases of the Tec family, like Akt and Btk, which are required to activate phospholipase C (PLC) [69, 70].

### 2.4. CD200R, a Receptor That Inhibits Activation of Macrophages and Propitiates the Survival of Intracellular Pathogens

#### 2.4.1. Structure and Activation Mechanism

**Figure 4:**
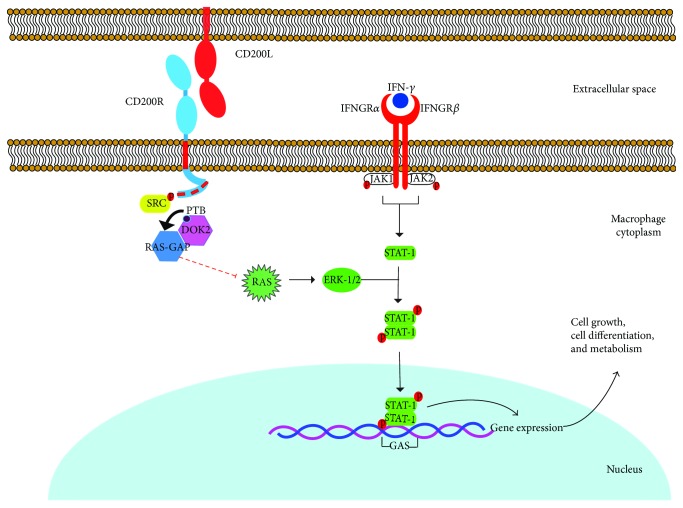
CD200R-induced signaling pathway. Interaction of CD200R with its ligand induces phosphorylation of the tyrosine residues (red squares) present in the intracellular portion of the receptor mediated by kinases of the Src family. This mechanism propitiates recruitment of Dok2 through its binding domain to tyrosine (PTB). Dok2 binds to the phosphorylated tyrosines, and it recruits the activator protein of Ras-GTPase (RasGAP), which inhibits Ras and downstream ERK activation. ERK is a MAP kinase which is involved in macrophages activation by IFN-*γ*. JAK/STAT-1 signaling pathway is required for cell activation, and STAT-1 is phosphorylated by JAK proteins and ERK-1/2 in turn to allow the gene expression for cell differentiation, growth and metabolism, so then, if CD200R binds to its ligand CD200 it allows the inhibition of cell activation [74, 95].

CD200R is a glycoprotein belonging to the superfamily of the immunoglobulins that are expressed on the surface of myeloid cells, principally in the subpopulation of regulator macrophages with the M2a phenotypical profile, which can be used as a specific marker [71, 72]. The extracellular portion of CD200R consists of two domains of immunoglobulin type (IgV and IgC2) [73]. This receptor contains a single transmembrane portion, which means that it can only cross the cell membrane through this region [73] (Figure 1). Unlike other inhibitory receptors, CD200R does not contain an ITIM domain in its intracytoplasmic tail; instead, studies have identified three tyrosine residues that are important for the function of CD200R (Y291, Y294, and Y302 in humans and Y286, Y289, and Y297 in mice). It has been shown that the interaction of CD200R with its ligand, CD200, in macrophages induces phosphorylation of tyrosine residues present in the intracellular portion of CD200R, a phosphorylation mediated by kinases of the Src family. The phosphorylation of these residues results in inducing recruitment of the adaptor protein Dok2 through its binding domain to the phosphorylated tyrosine (PTB), which then initiates the cascade of inhibitory signaling [74]. Once Dok2 has bound to the intracellular portion of CD200R, it can recruit other proteins, such as the activator protein of Ras-GTPase (RasGAP) [74]. In human macrophages, studies have shown that the recruitment and ensuing activation of RasGAP are essential for inhibiting the signaling pathway of the Ras-ERK and PI3K kinases [74] which, in turn, are essential for diverse, vital processes, including cell growth, differentiation, proliferation, and metabolism by activation of other transcriptional factor such as STAT-1 which also is involved in macrophage activation by IFN-*γ* [75]. This mechanism that regulates or inhibits activation in myeloid cells is clearly distinct from that of the receptors that contain ITIM motifs, which recruit phosphatases and are the principle effectors of the inhibition mediated by this type of receptor [76] (Figure 4).

Several studies have demonstrated that the interaction of CD200 with CD200R negatively regulates the activation of myeloid cells. This regulation may be caused by posttranslational modifications, such as tyrosine phosphorylation in the CD200R cytoplasmic tail, as well as increased expression of the receptor itself and its ligand CD200 in endothelial, epithelial and lymphoid cells, fibroblasts, and astrocytes [77, 78]. Also, it has been demonstrated that the expression of CD200 is induced by several nuclear factors such as NF-*κ*B(p65), STAT1a, and IRF-1 when they bind to their corresponding *cis*-elements which are found in the CD200 promoter region. Furthermore, one of these factors is c-Rel, the NF-*κ*B transcription factor, which is required for TLR-induced upregulation of CD200, probably induced by pathogens [77, 79]. Recently, it was determined that CD200R1 expression is regulated by C/EBPb and that overexpression of this transcription factor due to stimulation of microglia cells by LPS significantly reduces the expression of this receptor at the protein and mRNA level, but in the case of C/EBPb KO cells, this decrease was not seen [80]. The authors of that study suggested that the mechanism which inhibited CD200R expression was histone deacetylation since C/EBPb interacts with the histone deacetylase HDAC1 which, in turn, can bind directly to the CD200R promoter to inhibit transcription of the gene [80].

It has been reported that CD200R modulates the activation of cells in the microglia under conditions of acute, chronic inflammation by interacting with its ligand CD200 [81]. At the same time, CD200R expression is modulated by IL-4 [81]. An *in vivo* study with mice demonstrated that upon decreasing CD200R expression, activation of the cells in the microglia increases. CD200R expression decreased in IL-4KO mice, while stimulation with this cytokine increased expression of the receptor. These results are manifested in the macrophages of WT and IL-4 KO mice stimulated with LPS, as the latter showed higher production of the proinflammatory cytokines IL-1, IL-6, and TNF-*α*, and of the expression of CD40, while CD200R expression was null [82].

### 2.4.2. CD200 and Its Participation in Pathogenic Infections

*(1) Parasites*. In infections caused by intracellular parasites, cells increase their expression of CD200R and its ligand, but this has deleterious effects on the inflammatory response. For example, in wild-type mice infected with *Toxoplasma gondii*, research has documented the overexpression of CD200R in cells in the microglia and of CD200 in endothelial cells. This increase in CD200R expression has been associated with reduced cell activation and the production of molecules that are important in the immune response, such as TNF-*α*, iNOS, and MHC-II [83]. In contrast, in CD200 KO mice, it has been documented that the cells of the microglia increase their proliferation and activation rates, as well as the expression of MHC-II, TNF-*α*, and iNOS during chronic *Toxoplasma gondii* infection [78]. There is also evidence of a reduction in the parasitic load and mortality compared to WT mice that might be explained by the fact that the CD200 KO mice exhibit an increased inflammatory phenotype in response to ligands of TLRs, as shown by the increase in the production of TNF-*α* and IL-6, and the activation of the NF-*κ*B pathway [78].

A mouse model of parasitic infection of bone marrow macrophages with *Leishmania amazonensis* (*L. amazonensis*) has also made it possible to evaluate the role of CD200/CD200R. It has been identified that infections by this parasite induce increased expression of CD200 at the protein and mRNA level compared to uninfected macrophages [84]. It is not clear yet, but this increase in CD200 expression favors survival of the pathogen, perhaps because CD200 affects normal Th1 lymphocyte development which is essential for immune response against Leishmania; in fact, IFN-*γ*-activated macrophages produce NO to kill intracellular Leishmania major, but if CD200 is increased, it can avoid this immune response which can lead to the development of systemic Leishmaniasis [84]. Infected macrophages treated with iNOS inhibitors also foster replication of the pathogen and increase CD200 expression, suggesting, at least concerning *L. amazonensis* infections, that the infected macrophages can inhibit activation of neighboring macrophages by expressing both CD200 and CD200R.

In addition to the aforementioned mechanisms in which CD200/CD200R can regulate cell activation and the response to intracellular pathogens, another mechanism has been described through which infected macrophages can regulate the activation of other cells without any direct interaction. This is the release of exosomes and small vesicles (30–150 nm) that contain membrane proteins and provide signals to naive macrophages. This type of regulation was described recently; [85] hence, it is likely that the exosomes contain the CD200 protein and that, once secreted, the CD200 on their surface could bond to its receptor in neighboring macrophages with no need for cell-cell interaction.

*(2) Bacteria.* Aside from parasites, another pathogen that induces CD200 expression in macrophages in bone marrow is *Neisseria meningitidis*, through recognition of LPS by TLR4. A study in 2010 demonstrated that CD200 : CD200R interaction in WT mice negatively regulates the immune response against *N. meningitidis*. It has been documented that CD200 and CD200R expression is modified differentially when administered to macrophages of mice TLR agonists and for the inflammasome activators NOD2 and NALP3. That work showed that induction of CD200 expression is dependent on the c-Rel transcription factor and is negatively regulated when there is activation of the pathways activated through TLR and NOD2 [86]. This response turned out to be dependent on MyD88, but independent of the scavenger receptor A (SRA), which is considered one of the principle receptors that permits the interaction of macrophages with *Neisseria meningitidis* [87]. Additionally, agonists of pattern-recognizing receptors, such as NALP3, also induced CD200 expression, with c-Rel a member of transcription factor NF-*κ*B—being essential in this signaling pathway. In contrast, an increase in the mortality of CD200 KO mice was observed in response to experimental meningococcal septicemia, due to high levels of proinflammatory cytokines and activated leucocytes [86]. Infection by *Chlamydia trachomatis* also increased CD200R expression in macrophages of the endometrium and CD200 expression in epithelial cells from the genital tract by regulating inflammation and suppressing collateral damage in the tissue during chronic infection, thus enabling persistence [88]. These mechanisms are consistent with the absence of changes in the vaginal epithelium, which complicates clinical studies of infected patients. It has also been reported that infection with *Chlamydia* increases the percentage of macrophages in the endometrium that coexpress CD200R and CD206 (mannose receptor), both of which are markers of alternately activated macrophages (M2) [88].

*(3) Virus*. Models of virus infections have also documented the importance of the CD200/CD200R pathway and its role as a regulator of the immune response. For example, it has been shown that CD200 KO mice infected with the influenza A virus manifest greater weight loss and higher mortality rates than native mice. These effects may cause the increase in NO levels in the lung and of the cytokines IL-6, TNF-*α*, IFN-*γ*, and MIP-1*α* measured in bronchoalveolar lavage [89]. In contrast, administering the anti-CD200R agonist (CD200-Fc) partially reverses the phenotype of CD200 KO mice by reducing weight loss and the number of cells compared to KO mice not treated with this agonist [90]. In this case, the function of CD200/CD200R interaction is to protect the host from “the cytokine storm” produced in response to the infection, which is the principal cause of the deleterious effects seen in CD200 KO mice.

In a 2011 study, Goulding et al. used an *in vivo* mouse model to show that the absence of the CD200R receptor in macrophages initially infected with the influenza virus and later with *Streptococcus pneumoniae* reduced the bacterial load and prevented mortality in WT mice [91]. That study suggested that the absence of CD200R is beneficial for the host because the exacerbated inflammation that occurred in the WT mice contributed to disease pathogenesis and increased the viral load [91]. In this sense, the inhibition or attenuation of cell activation by blocking the CD200R receptor would provide an advantage for treating secondary infections by counteracting the exacerbated inflammatory responses that might occur in such infections.

In another approach, Soberman et al. used a mouse model of encephalitis by the herpes simple virus (HSV-1) infection to demonstrate that increased morbidity and mortality that had been seen previously were related with the release of cytokines and chemokines that occurred once virus was recognized by TLR2. In contrast, in CD200 KO mice, observations showed a reduced inflammatory response against HSV-1, since the production of cytokines, such as IFN-*γ*, IL-6, and CCL5 (RANTES) decreased by 80% compared to WT mice [92]. However, the WT and CD200R KO mice did not differ in terms of IL-1*β* production, and in both types, the study detected adequate activation of inflammasome after stimulation with LPS and ATP [92]. This suggests that the pathway mediated by the CD200R receptor in mouse macrophages does not interfere with the activation of inflammasome and does not affect the production and release of IL-1*β*. Hence, we can consider that the CD200R receptor can regulate the activation mediated by membrane receptors for PAMPs, such as TLR4, but does not affect receptors of the NOD family. An additional way in which virus utilize the CD200/CD200R signaling pathway to counteract the host's inflammatory response is by incorporating into its genome proteins that are orthologues of the CD200 in the target cell. One of the most characteristic genes that codifies these orthologue proteins is the K14 gene of herpes 8 virus (HHV8), better known as the Kaposi sarcoma-associated herpes virus, which codifies a viral orthologue of CD200 (vOX2), whose expression on the surface of infected cells occurs during the lytic phase. Although vOX2 shares 36–40% of the identity of CD200 in humans, it bonds with similar affinity to its ligand and negatively regulates the release of TNF-*α*, G-CSF, and MCP-1 from macrophages activated with IFN-*γ* and LPS [93]. In addition to this protein, there is another viral orthologue of CD200: the R15 protein of the adenovirus that infects rhesus monkeys (RRV) and is a gamma herpes virus similar to HHV8. This protein is expressed on the surface of infected cells and is released into the supernatant of cell cultures. R15 reduces mRNA expression and the release of TNF-*α* from THP-1 macrophages activated with PMA, as well as the primary monocytes and macrophages of rhesus monkeys. These levels of inhibition were similar to those caused by CD200 in humans [94]. Therefore, these proteins of viral origin provide new therapeutic targets for which we can design and synthesize compounds to be directed against viral products of this kind to eliminate, or counteract, infection or viral propagation.

It is clear that diverse pathogens utilize, for their benefit, the signaling pathway realized by CD200/CD200R interaction to limit the inflammatory response and so survive inside the host cell. However, few studies have focused on identifying the molecules that are associated with this signaling. Thus, understanding how infectious agents use this pathway to regulate the host's defenses may lead to the development of new clinical tools and therapeutic strategies. Depending on the context of the infection or pathology, a blocking antibody, for example, directed against CD200R to block its interaction with CD200, could be effective in counteracting infections by pathogens whose survival is favored by, or associated with, overexpression of CD200R, complemented by conventional antimicrobial treatment. In contrast, using agonists to activate the CD200/CD200R signaling pathway, or adjacent cells, may also contribute to inducing the effector immune response.

## 3. Conclusions

The nature of the innate mechanisms of the immune response has become increasingly complex. Immunologists used to speak of how these mechanisms, made up of cells and receptors with some soluble elements, functioned through nonspecific recognition, in contrast to adaptive immunity mechanisms. Today, we know that all proteins that participate in pathogen recognizing perform this task, in a pathogen-associated molecular patterns recognition dependent way. This recognition of patterns indicates that nonspecific recognition by the innate immune response does not exist but we consider it as nonspecific because the variety of antigens recognized is limited when compared to adaptive mechanisms. Something similar occurs with the regulation of activation. While we do not yet know exactly how many copies of an antigen must enter an organism to trigger activation of specific lymphocytes, we do know that the activation signals generated by the presence of these antigens must cross a threshold—or limit—in order to emit activation signals that are sufficiently intense to trigger a successful activation process. Also, the intensity of the activation signal must exeed the signal given by the inhibitory receptors present in the cells of immune system that, as mentioned earlier, participate in diverse signaling mechanisms and pathways whose purpose is to prevent an exacerbated, or even unnecessary, activation process.

This text has focused on four receptors that are known to have opposite functions to the receptors that mediate cell activation. TIM-3, PD-1, CD-32b, and CD200R form a group of receptors that use diverse mechanisms to inhibit cell activation, such as association with phosphatases or steric impedance in the plasmatic membrane. However, there are other receptors like SIRP-*α*/CD47, which have similar role and function recruiting phosphatases as CD32b. There is reliable evidence that the balance between activation and inhibition signals also occurs in cells with innate functions, such as monocytes and macrophages, suggesting that the function and activation of these cells are a highly regulated process in which it has often been demonstrated that the loss of regulation can generate such deleterious processes as exacerbated inflammation or the loss of tolerance to autoantigens. Several authors refer to these regulating mechanisms as response elements that are capable of generating immunological tolerance, although others consider that we are dealing with mechanisms that inhibit activation which, when functioning at a high level, cause a dysfunctional state in immune cells. In reality, we should consider that no such frontier exists between these two descriptions but that we are trying to define the extremes of a concept that consists of a broad range of levels of regulation, the lowest of which is inhibition. The level associated with exacerbated responses and the opposite level of regulation would be the one that, as an overinduced mechanism, causes lack of cell response or cell senescence upon recognizing an antigen. Given all the possible immunological scenarios that can emerge from the presence or absence of inhibitory receptors and their functions, it is extremely important to continue studying these proteins in both lymphoid and myeloid cells. Obtaining a thorough understanding of the function of these receptors may provide new ways of dealing, clinically, with diverse pathologies and of increasing the effectiveness of current treatments.

## Abbreviations

Balb/c: Strain of laboratory-raised albino mice
BAT-3: Lymphocyte-associated transcript 3
BCR: B cell receptors
C/EBPb: Bonding beta protein to potentiator/CCAAT
CD: Differentiation group
ERK: Kinases regulated by extracellular signaling
Fc: Crystallizable region of immunoglobulin
FcR: Receptor of the crystallizable region of immunoglobulin
Gal-9: Galectin-9
GAS: Activation site of IFN-*γ*
G-CSF: Stimulating factor of granulocytic colonies
HBA: Hepatitis B virus
HCV: Hepatitis C virus
HDAC1: Deacetylase 1
HHV8: Human herpes 8 virus
HSV-1: Herpes simple 1 virus
IFN: Interferon gamma
IgC: Constant region of immunoglobulin
IgG: Immunoglobulin G
IgGR: Immunoglobulin G receptor
IgV: Variable region of immunoglobulin
IL: Interleukin
iNOS: Nitrous oxide inducible synthetase
ITAM: Tyrosine-associated activation motifs
ITIM: Tyrosine-associated inhibition motifs
ITK: Tyrosine kinase inhibitor
ITSM: Tyrosine-associated change motifs
JAK: Janus kinase
KO: Knockout
Lck: Leucocyte-specific tyrosine kinase protein
LPS: Lipopolysaccharide
M2a: Regulator macrophages
MAL: MyD88-type adapter
MHC: Principle histocompatibility compound
MIP-1*α:*: Alpha 1 macrophage inducible protein
mRNA: Messenger ribonucleic acid
MyD88: Response gene 88 to myeloid differentiation
NF-*κ*B: Nuclear factor kappa B
NK: Killer cell
NLRP3: NOD-associated protein
NO: Nitrous oxide
NOD2: Nucleotide 2 oligomerization domain
PD-1: Cell death protein
PDL1: PD-1 ligand
PI3K: Phosphatidyl inositol kinase
PIP3: Phosphatidyl inositol triphosphate
PLC: Phospholipase C
PMA: Myristyl phosphate acetate
Ps: Phosphatidylserine
PTB: Bonding domain to phosphorylated tyrosine
RasGAP: Activator protein of Ras-GTPase
RRV: Virus of the rhesus monkey, family *adenoviridae*
SHIP: Phosphatase 1 of inositol containing the SH2 domain
SHP: Tyrosine phosphatase containing the residue of homology 2 of Src proteins
siRNA: Interference ribonucleic acid
SRA: Scavenger receptor A
STAT: Signal transducer and activator of transcription 3
Tc1: Cytotoxic T lymphocytes
TCR: T cell receptor
Th1: T lymphocyte cooperator 1
THP-1: Human monocyte cell line from acute monocytic leukemia
TIM-3: Protein of family 3 of T lymphocytes containing mucin- and immunoglobulin-type domains
TIR: Homologous region to IL-1R/Toll
TLR: Toll-type receptor
TNF-*α:*: Alpha tumor necrosis factor
TRAM: TRIF-related adaptor molecule
TRIFF: Adaptor with TIR domain, beta interferon inducer
vOX2: Viral orthologue of CD200
WT: Wild-type.

## References

[1] Wood S., Jayaraman V., Huelsmann E. J. (2014). Pro-inflammatory chemokine CCL2 (MCP-1) promotes healing in diabetic wounds by restoring the macrophage response. *PLoS One*.

[2] Singh S., Barr H., Liu Y. C. (2015). Granulocyte-macrophage colony stimulatory factor enhances the pro-inflammatory response of interferon-γ-treated macrophages to Pseudomonas aeruginosa infection. *PLoS One*.

[3] Gordon S. (2016). Phagocytosis: an immunobiologic process. *Immunity*.

[4] Ginhoux F., Guilliams M. (2016). Tissue-resident macrophage ontogeny and homeostasis. *Immunity*.

[5] Hashimoto D., Chow A., Noizat C. (2013). Tissue-resident macrophages self-maintain locally throughout adult life with minimal contribution from circulating monocytes. *Immunity*.

[6] Gordon S., Plüddemann A. (2017). Tissue macrophages : heterogeneity and functions. *BMC Biology*.

[7] Gordon S. (2003). Alternative activation of macrophages. *Nature Reviews Immunology*.

[8] Martinez F. O., Gordon S. (2014). The M1 and M2 paradigm of macrophage activation : time for reassessment. *F1000Prime Reports*.

[9] Gonzalez N. A., Quintana J. A., García-Silva S. (2017). Phagocytosis imprints heterogeneity in tissue-resident macrophages. *Journal of Experimental Medicine*.

[10] He Q., Johnston J., Zeitlinger J. (2015). ChIP-nexus enables improved detection of *in vivo* transcription factor binding footprints. *Nature Biotechnology*.

[11] Kimura T., Sakamoto H., Appella E., Siraganian R. P. (1996). Conformational changes induced in the protein tyrosine kinase p72syk by tyrosine phosphorylation or by binding of phosphorylated immunoreceptor tyrosine-based activation motif peptides. *Molecular and Cellular Biology*.

[12] Ivashkiv L. B. (2009). Cross-regulation of signaling by ITAM-associated receptors. *Nature Immunology*.

[13] Schneider H., Prasad K. V., Shoelson S. E., Rudd C. E. (1995). CTLA-4 binding to the lipid kinase phosphatidylinositol 3-kinase in T cells. *Journal of Experimental Medicine*.

[14] Karlhofer F. M., Ribaudo R. K., Yokoyama W. M. (1992). MHC class I alloantigen specificity of Ly-49^+^ IL-2-activated natural killer cells. *Nature*.

[15] Zhu Y., Yao S., Chen L. (2011). Cell surface signaling molecules in the control of immune responses: a tide model. *Immunity*.

[16] Long E. O. (1999). Regulation of immune responses through inhibitory receptors. *Annual Review of Immunology*.

[17] Muta T., Kurosaki T., Misulovin Z., Sanchez M., Nussenzweig M. C., Ravetch J. V. (1994). A 13-amino-acid motif in the cytoplasmic domain of FcγRIIB modulates B-cell receptor signalling. *Nature*.

[18] Bruhns P., Vély F., Malbec O., Fridman W. H., Vivier E., Daëron M. (2000). Molecular basis of the recruitment of the SH2 domain-containing inositol 5-phosphatases SHIP1 and SHIP2 by FcγRIIB. *The Journal of Biological Chemistry*.

[19] Fuertes Marraco S. A., Neubert N. J., Verdeil G., Speiser D. E. (2015). Inhibitory receptors beyond T cell exhaustion. *Frontiers in Immunology*.

[20] Maeda A., Kurosaki M., Ono M., Takai T., Kurosaki T. (1998). Requirement of SH2-containing protein tyrosine phosphatases SHP-1 and SHP-2 for paired immunoglobulin-like receptor B (PIR-B)–mediated inhibitory signal. *The Journal of Experimental Medicine*.

[21] Odorizzi P. M., Wherry E. J. (2012). Inhibitory receptors on lymphocytes: insights from infections. *The Journal of Immunology*.

[22] Butte M. J., Keir M. E., Phamduy T. B., Sharpe A. H., Freeman G. J. (2007). PD-L1 interacts specifically with B7-1 to inhibit T cell proliferation. *Immunity*.

[23] Egen J. G., Kuhns M. S., Allison J. P. (2002). CTLA-4: new insights into its biological function and use in tumor immunotherapy. *Nature Immunology*.

[24] Long E. O. (2008). Negative signaling by inhibitory receptors: the NK cell paradigm. *Immunological Reviews*.

[25] Moser J. M., Gibbs J., Jensen P. E., Lukacher A. E. (2002). CD94-NKG2A receptors regulate antiviral CD8^+^ T cell responses. *Nature Immunology*.

[26] Lin G. G., Scott J. G. (2011). Investigations of the constitutive overexpression of *CYP6D1* in the permethrin resistantLPR strain of house fly (*Musca domestica*). *Pesticide Biochemistry and Physiology*.

[27] Ishida Y., Agata Y., Shibahara K., Honjo T. (1992). Induced expression of PD-1, a novel member of the immunoglobulin gene superfamily, upon programmed cell death. *The EMBO Journal*.

[28] Dong H., Zhu G., Tamada K., Chen L. (1999). B7-H1, a third member of the B7 family, co-stimulates T-cell proliferation and interleukin-10 secretion. *Nature Medicine*.

[29] Latchman Y., Wood C. R., Chernova T. (2001). PD-L2 is a second ligand for PD-1 and inhibits T cell activation. *Nature Immunology*.

[30] Jin H., Anderson A. C., Tan W. G. (2010). Cooperation of Tim-3 and PD-1 in CD8 T-cell exhaustion during chronic viral infection. *Proceedings of the National Academy of Sciences of the United States of America*.

[31] Sakuishi K., Apetoh L., Sullivan J. M., Blazar B. R., Kuchroo V. K., Anderson A. C. (2010). Targeting Tim-3 and PD-1 pathways to reverse T cell exhaustion and restore anti-tumor immunity. *The Journal of Experimental Medicine*.

[32] Brahmer J. R., Drake C. G., Wollner I. (2010). Phase I study of single-agent anti-programmed death-1 (MDX-1106) in refractory solid tumors: safety, clinical activity, pharmacodynamics, and immunologic correlates. *Journal of Clinical Oncology*.

[33] Benson D. M., Bakan C. E., Mishra A. (2010). The PD-1/PD-L1 axis modulates the natural killer cell versus multiple myeloma effect: a therapeutic target for CT-011, a novel monoclonal anti-PD-1 antibody. *Blood*.

[34] Hamid O., Robert C., Daud A. (2013). Safety and tumor responses with lambrolizumab (anti-PD-1) in melanoma. *The New England Journal of Medicine*.

[35] Ma C. J., Ni L., Zhang Y. (2011). PD-1 negatively regulates interleukin-12 expression by limiting STAT-1 phosphorylation in monocytes/macrophages duringchronic hepatitis C virus infection. *Immunology*.

[36] Cho H. Y., Choi E. K., Lee S. W. (2009). Programmed death-1 receptor negatively regulates LPS-mediated IL-12 production and differentiation of murine macrophage RAW264.7 cells. *Immunology Letters*.

[37] Zhang Y., Ma C. J., Wang J. M. (2011). Tim-3 negatively regulates IL-12 expression by monocytes in HCV infection. *PLoS One*.

[38] Zhang Z. N., Zhu M. L., Chen Y. H. (2015). Elevation of Tim-3 and PD-1 expression on T cells appears early in HIV infection, and differential Tim-3 and PD-1 expression patterns can be induced by common *γ*-chain cytokines. *BioMed Research International*.

[39] Gordon S. R., Maute R. L., Dulken B. W. (2017). PD-1 expression by tumour-associated macrophages inhibits phagocytosis and tumour immunity. *Nature*.

[40] Ansell S. M., Lesokhin A. M., Borrello I. (2015). PD-1 blockade with nivolumab in relapsed or refractory Hodgkin’s lymphoma. *The New England Journal of Medicine*.

[41] Qu Q. X., Huang Q., Shen Y., Zhu Y. B., Zhang X. G. (2016). The increase of circulating PD-L1-expressing CD68^+^ macrophage in ovarian cancer. *Tumor Biology*.

[42] Dannenmann S. R., Thielicke J., Stöckli M. (2013). Tumor-associated macrophages subvert T-cell function and correlate with reduced survival in clear cell renal cell carcinoma. *OncoImmunology*.

[43] Monney L., Sabatos C. A., Gaglia J. L. (2002). Th1-specific cell surface protein Tim-3 regulates macrophage activation and severity of an autoimmune disease. *Nature*.

[44] Chiba S., Baghdadi M., Akiba H. (2012). Tumor-infiltrating DCs suppress nucleic acid-mediated innate immune responses through interactions between the receptor TIM-3 and the alarmin HMGB1. *Nature Immunology*.

[45] Shang Y., Li Z., Li H., Xia H., Lin Z. (2013). TIM-3 expression in human osteosarcoma: correlation with the expression of epithelial-mesenchymal transition-specific biomarkers. *Oncology Letters*.

[46] Kadowaki T., Arikawa T., Shinonaga R. (2012). Galectin-9 signaling prolongs survival in murine lung-cancer by inducing macrophages to differentiate into plasmacytoid dendritic cell-like macrophages. *Clinical Immunology*.

[47] Cao Y., Zhou X., Huang X. (2013). Tim-3 expression in cervical cancer promotes tumor metastasis. *PLoS One*.

[48] Ma C. J., Li G. Y., Cheng Y. Q. (2013). *Cis* association of galectin-9 with Tim-3 differentially regulates IL-12/IL-23 expressions in monocytes via TLR signaling. *PLoS One*.

[49] DeKruyff R. H., Bu X., Ballesteros A. (2010). T cell/transmembrane, Ig, and mucin-3 allelic variants differentially recognize phosphatidylserine and mediate phagocytosis of apoptotic cells. *The Journal of Immunology*.

[50] Lee M. J., Heo Y. M., Hong S., Kim K., Park S. (2009). The binding properties of glycosylated and non-glycosylated Tim-3 molecules on CD4^+^CD25^+^ T cells. *Immune Network*.

[51] Lee J., Su E. W., Zhu C. (2011). Phosphotyrosine-dependent coupling of tim-3 to T-cell receptor signaling pathways. *Molecular and Cellular Biology*.

[52] deCathelineau A. M., Henson P. M. (2003). The final step in programmed cell death: phagocytes carry apoptotic cells to the grave. *Essays in Biochemistry*.

[53] Kobayashi N., Karisola P., Peña-Cruz V. (2007). TIM-1 and TIM-4 glycoproteins bind phosphatidylserine and mediate uptake of apoptotic cells. *Immunity*.

[54] Cao E., Zang X., Ramagopal U. A. (2007). T cell immunoglobulin mucin-3 crystal structure reveals a Galectin-9-independent ligand-binding surface. *Immunity*.

[55] Nakayama M., Akiba H., Takeda K. (2009). Tim-3 mediates phagocytosis of apoptotic cells and cross-presentation. *Blood*.

[56] Asano K., Miwa M., Miwa K. (2004). Masking of phosphatidylserine inhibits apoptotic cell engulfment and induces autoantibody production in mice. *The Journal of Experimental Medicine*.

[57] Zhu C., Anderson A. C., Schubart A. (2005). The Tim-3 ligand galectin-9 negatively regulates T helper type 1 immunity. *Nature Immunology*.

[58] Golden-Mason L., Palmer B. E., Kassam N. (2009). Negative immune regulator Tim-3 is overexpressed on T cells in hepatitis C virus infection and its blockade rescues dysfunctional CD4^+^ and CD8^+^ T cells. *Journal of Virology*.

[59] Bezbradica J. S., Schroder K. (2014). TRAF6 is a nexus for TLR-STAT1 crosstalk. *Immunology and Cell Biology*.

[60] Luu K., Greenhill C. J., Majoros A., Decker T., Jenkins B. J., Mansell A. (2014). STAT1 plays a role in TLR signal transduction and inflammatory responses. *Immunology and Cell Biology*.

[61] Zhang Y., Ma C. J., Wang J. M. (2012). Tim-3 regulates pro- and anti-inflammatory cytokine expression in human CD14^+^ monocytes. *Journal of Leukocyte Biology*.

[62] Chen Y., Hsieh M. Y., Chang M. Y. (2012). Eps8 protein facilitates phagocytosis by increasing TLR4-MyD88 protein interaction in lipopolysaccharide-stimulated macrophages. *Journal of Biological Chemistry*.

[63] Gong P., Angelini D. J., Yang S. (2008). TLR4 signaling is coupled to SRC family kinase activation, tyrosine phosphorylation of zonula adherens proteins, and opening of the paracellular pathway in human lung microvascular endothelia. *Journal of Biological Chemistry*.

[64] Ernst L. K., Duchemin A. M., Anderson C. L. (1993). Association of the high-affinity receptor for IgG (Fc gamma RI) with the gamma subunit of the IgE receptor. *Proceedings of the National Academy of Sciences of the United States of America*.

[65] Brooks D., Qiu W., Luster A., Ravetch J. V. (1989). Structure and expression of human IgG FcRII(CD32) functional heterogeneity is encoded by the alternatively spliced products of multiple genes. *The Journal of Experimental Medicine*.

[66] Van den Herik-Oudijk I. E., Capel P. J., van der Bruggen T., Van de Winkel J. G. (1995). Identification of signaling motifs within human Fc gamma RIIa and Fc gamma RIIb isoforms. *Blood*.

[67] Jensen W. A., Marschner S., Ott V. L., Cambier J. C. (2001). FcγRIIB-mediated inhibition of T-cell receptor signal transduction involves the phosphorylation of SH2-containing inositol 5-phosphatase (SHIP), dephosphorylation of the linker of activated T-cells (LAT) and inhibition of calcium mobilization. *Biochemical Society Transactions*.

[68] Ono M., Bolland S., Tempst P., Ravetch J. V. (1996). Role of the inositol phosphatase SHIP in negative regulation of the immune system by the receptor Fc(gamma)RIIB. *Nature*.

[69] Wang Y., Wu Y., Wang Z. (2006). Akt binds to and phosphorylates phospholipase C-*γ*1 in response to epidermal growth factor. *Molecular Biology of the Cell*.

[70] Bolland S., Pearse R. N., Kurosaki T., Ravetch J. V. (1998). SHIP modulates immune receptor responses by regulating membrane association of Btk. *Immunity*.

[71] Caserta S., Nausch N., Sawtell A. (2012). Chronic infection drives expression of the inhibitory receptor CD200R, and its ligand CD200, by mouse and human CD4 T cells. *PLoS One*.

[72] Wright G. J., Puklavec M. J., Willis A. C. (2000). Lymphoid/neuronal cell surface OX2 glycoprotein recognizes a novel receptor on macrophages implicated in the control of their function. *Immunity*.

[73] Hatherley D., Lea S. M., Johnson S., Barclay A. N. (2013). Structures of CD200/CD200 receptor family and implications for topology, regulation, and evolution. *Structure*.

[74] Mihrshahi R., Barclay A. N., Brown M. H. (2009). Essential roles for Dok2 and RasGAP in CD200 receptor-mediated regulation of human myeloid cells. *The Journal of Immunology*.

[75] Josephs D. H., Sarker D. (2016). Pharmacodynamic biomarker development for PI3K pathway therapeutics. *Translational Oncogenomics*.

[76] Daëron M., Jaeger S., Du Pasquier L., Vivier E. (2008). Immunoreceptor tyrosine-based inhibition motifs: a quest in the past and future. *Immunological Reviews*.

[77] Chen Z., Marsden P. A., Gorczynski R. M. (2009). Role of a distal enhancer in the transcriptional responsiveness of the human CD200 gene to interferon-*γ* and tumor necrosis factor-*α*. *Molecular Immunology*.

[78] Costello D. A., Lyons A., Denieffe S., Browne T. C., Cox F. F., Lynch M. A. (2011). Long term potentiation is impaired in membrane glycoprotein CD200-deficient mice: a role for toll-like receptor activation. *The Journal of Biological Chemistry*.

[79] Rosenblum M. D., Olasz E., Woodliff J. E. (2004). CD200 is a novel p53-target gene involved in apoptosis-associated immune tolerance. *Blood*.

[80] Dentesano G., Straccia M., Ejarque-Ortiz A. (2012). Inhibition of CD200R1 expression by C/EBP beta in reactive microglial cells. *Journal of Neuroinflammation*.

[81] Lyons A., McQuillan K., Deighan B. F. (2009). Decreased neuronal CD200 expression in IL-4-deficient mice results in increased neuroinflammation in response to lipopolysaccharide. *Brain, Behavior, and Immunity*.

[82] Lyons A., Griffin R. J., Costelloe C. E., Clarke R. M., Lynch M. A. (2007). IL-4 attenuates the neuroinflammation induced by amyloid-beta in vivo and in vitro. *Journal of Neurochemistry*.

[83] Deckert M., Sedgwick J. D., Fischer E., Schlüter D. (2006). Regulation of microglial cell responses in murine toxoplasma encephalitis by CD200/CD200 receptor interaction. *Acta Neuropathologica*.

[84] Cortez M., Huynh C., Fernandes M. C., Kennedy K. A., Aderem A., Andrews N. W. (2011). Leishmania promotes its own virulence by inducing expression of the host immune inhibitory ligand CD200. *Cell Host & Microbe*.

[85] Bhatnagar S., Shinagawa K., Castellino F. J., Schorey J. S. (2007). Exosomes released from macrophages infected with intracellular pathogens stimulate a proinflammatory response in vitro and in vivo. *Blood*.

[86] Mukhopadhyay S., Plüddemann A., Claire Hoe J. (2010). Immune inhibitory ligand CD200 induction by TLRs and NLRs limits macrophage activation to protect the host from meningococcal septicemia. *Cell Host & Microbe*.

[87] Peiser L., Makepeace K., Pluddemann A. (2006). Identification of neisseria meningitidis nonlipopolysaccharide ligands for class A macrophage scavenger receptor by using a novel assay. *Infection and Immunity*.

[88] Vicetti Miguel R. D., Harvey S. A. K., LaFramboise W. A., Reighard S. D., Matthews D. B., Cherpes T. L. (2013). Human female genital tract infection by the obligate intracellular bacterium chlamydia trachomatis elicits robust type 2 immunity. *PLoS One*.

[89] Karnam G., Rygiel T. P., Raaben M. (2012). CD200 receptor controls sex-specific TLR7 responses to viral infection. *PLoS Pathogens*.

[90] Snelgrove R. J., Goulding J., Didierlaurent A. M. (2008). A critical function for CD200 in lung immune homeostasis and the severity of influenza infection. *Nature Immunology*.

[91] Goulding J., Godlee A., Vekaria S., Hilty M., Snelgrove R., Hussell T. (2011). Lowering the threshold of lung innate immune cell activation alters susceptibility to secondary bacterial superinfection. *The Journal of Infectious Diseases*.

[92] Soberman R. J., MacKay C. R., Vaine C. A. (2012). CD200R1 supports HSV-1 viral replication and licenses pro-inflammatory signaling functions of TLR2. *PLoS One*.

[93] Foster-Cuevas M., Westerholt T., Ahmed M., Brown M. H., Barclay A. N., Voigt S. (2011). Cytomegalovirus e127 protein interacts with the inhibitory CD200 receptor. *Journal of Virology*.

[94] Langlais C. L., Jones J. M., Estep R. D., Wong S. W. (2006). Rhesus rhadinovirus R15 encodes a functional homologue of human CD200. *Journal of Virology*.

[95] Zhang S., Cherwinski H., Sedgwick J. D., Phillips J. H. (2004). Molecular mechanisms of CD200 inhibition of mast cell activation. *The Journal of Immunology*.

